# 3D optical/CT as a preclinical companion imaging platform for glioblastoma drug development

**DOI:** 10.1080/10717544.2020.1833381

**Published:** 2020-12-02

**Authors:** Andrei Molotkov, Mikhail Doubrovin, Nikunj Bhatt, Fang-Chi Hsu, Amanda Beserra, Rajiv Chopra, Akiva Mintz

**Affiliations:** a Columbia University PET Center, Department of Radiology, Columbia University Medical Center, New York, NY, USA; b Department of Biostatistics and Data Science, Division of Public Health Sciences, Wake Forest School of Medicine, Winston-Salem, NC, USA; c Department of Radiology and Advanced Imaging Research Center, UT Southwestern Medical Center, Dallas, TX, USA

**Keywords:** Blood–brain barrier, glioblastoma, focused ultrasound, 3D optical imaging, PET

## Abstract

Multimodality 3D Optical Imaging (OI)/CT has the potential to play a major role in drug development for glioblastomas (GBM), as it is an accessible preclinical method. To demonstrate the potential of 3D OI/CT to visualize orthotopic GBM implantation, we labeled GBM cells with Cy7 and imaged their location using 3D OI/CT. To confirm the accuracy of the spatial localization and demonstrate the ability to image locoregionally delivered therapies, we labeled mouse albumin with Cy7 (Cy7ALB) and delivered it via locoregional infusion 1 mm or 3 mm into the brain and demonstrated correlation of signal between the 3D OI/CT and post necropsy brain slices. In addition, we demonstrated the potential of systemically delivered Cy7ALB contrast to detect blood–brain barrier (BBB) permeability caused by orthotopic GBMs using 3D OI/CT. We also tested the potential of 3D OI/CT to assess focal BBB permeability induced by high intensity focused ultrasound (HIFU), a methodology being used in clinical trials to noninvasively permeabilize the BBB for systemic therapeutic delivery to GBM. We demonstrated the ability of systemic Cy7ALB contrast together with 3D OI/CT to accurately assess real-time HIFU-induced BBB permeability, which correlated to post necropsy imaging of brains. Furthermore, we demonstrated that 3D OI/CT can also image the therapeutic distribution of a Cy7-labeled anti-PD-1 antibody, a prototype translational antibody therapy. We successfully imaged real-time antibody distribution after HIFU-induced BBB permeability, which correlated with post necropsy Cy7 signal and translational PET imaging after injection of [^89^Zr] anti-PD-1 antibody. Thus, we demonstrated the broad potential of using 3D OI/CT as an accessible preclinical tool to develop anti-GBM therapies.

## Introduction

Glioblastoma (GBM) is the most common primary adult brain tumor with an extremely poor prognosis and median survival of fewer than 2 years (Wen & Kesari, 2008; Preusser et al., 2015). One key reason for this high mortality is that the blood–brain barrier (BBB) significantly restricts the targeted delivery of therapeutics to brain tumors (Dyrna et al., 2013). Although the core of the tumor has a leaky BBB that allows MRI contrast to permeabilize and can therefore successfully be imaged, areas of tumor cell infiltration that exist outside this contrast enhancing center are not well permeabilized by systemically administered therapies and therefore, severely limit the number of drugs that can be effective against this deadly tumor. Convection enhanced delivery (CED) and high intensity focused ultrasound (HIFU) are 2 emerging delivery modalities that offer the promise of reaching the infiltrating GBM cells outside the BBB-permeable core. CED is a form of loco-regional delivery that involves infusing small volumes of therapeutic under pressure directly into the tumor and surrounding tissue using strategically placed catheters. CED has advanced significantly in recent years to include highly sophisticated catheter placement planning, advanced anti-backflow catheters, and novel multi-catheter arrays that perfuse the entire tumor volume (Debinski & Tatter, 2009, 2010; Debinski et al., 2017). HIFU is a non-invasive method of permeabilizing the BBB using ultrasound waves in conjunction with systemically administered microbubbles and has shown promise in preclinical and early-stage clinical trials (Alkins et al., 2013; Sattiraju, Sai, Xuan, et al., 2017).

However, in order to advance these methods and test the true efficacy of anti-GBM therapies, it is critical to developing higher throughput precise methods of evaluating preclinical tumor growth and location, evaluating BBB permeability, and evaluating drug distribution. While small animal MRI is the current gold standard in preclinical GBM imaging, it is not typically available or cost-effective for larger-scale experiments. This limitation has led many groups to test therapeutics by measuring survival, which is grossly imprecise because even using stereotactic tumor placement, tumors can form at locations even a millimeter apart and impinge on very different structures that independently have very different mortality rates. Furthermore, implanted tumor cells are notorious for growing outside the brain tissue, which can bypass the BBB and therefore no longer represents an orthotopic model of the human disease. Additionally, for testing new drugs that do not naturally cross the BBB, it is critical to ensure that the BBB is properly permeabilized in the correct location (HIFU), or the drug is successfully delivered via a properly placed and functioning catheter (CED).

**Figure 1.**
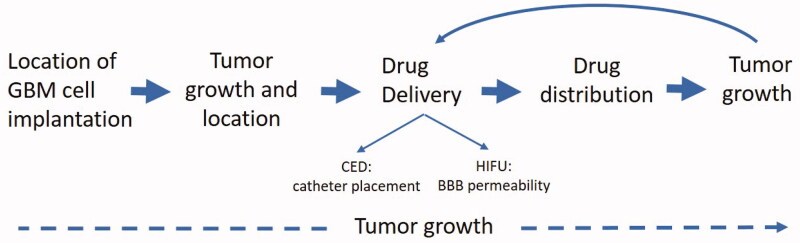
Uses of optical/CT along the preclinical translational spectrum. 3D OI/ CT imaging allows for precise localization of the intracranial implanted NIR-labeled GBM cells. After GBM cell implantation, 3D OI/CT imaging enables visualization of the tumor growth and intracranial location of the tumor. 3D OI/CT imaging using Cy7 labeled albumin validates BBB opening with HIFU or CED drug delivery allowing visualization of drug distribution.

Near-infrared (NIR) optical imaging is preferable to standard fluorescent imaging of green or red fluorescent proteins due to its decreased autofluorescence and background signals. Furthermore, NIR optical imaging has the advantage of deeper tissue penetration, enabling signal detection in all regions of small animals like mice. Optical scanners are typically equipped with lasers that penetrate tissue and excite specific NIR dyes with matching excitation wavelengths, allowing detection of NIR labeled molecules, peptides, and proteins in a live animal. Recent innovations in NIR imaging allows 3D Fluorescence Tomography (FLT) by using information obtained from an adjacent CT scanner for the reconstruction of 3D optical images. These 3D optical images, when fused to detailed anatomic images from CT, have the potential to localize labeled tumor cells, contrast, or drugs. Therefore, the objective of this work is to develop 3D OI/CT as an accessible, cost-effective modality of visualizing preclinical tumor cell placement, tumor growth, locoregional therapeutic administration, HIFU-mediated BBB permeability, and therapeutic distribution (Figure 1).

## Material and methods

### Cell culture

Firefly luciferase-expressing mouse glioblastoma cells derived from a retroviral model (Lei et al., 2011) were gifts from Dr. Peter Canoll (Columbia University) (Karpel-Massler et al., 2017). Firefly luciferase-expressing human glioblastoma (GBM) G48a cells (Debinski & Gibo, 2005) were cultured in DMEM basal media supplemented with 10% of heat-inactivated FBS (Gibco, Ireland), 2 mM glutamine, and 1/200 of the penicillin–streptomycin mix. Murine glioma cells were cultured in DMEM basal media supplemented with 0.5% FBS (Gibco, Ireland), 1/100 N2 supplement (Gibco, Ireland), 1/100 of anti/anti (Gibco, Ireland), and 10 ng/ml of heat-stable recombinant human bFGF (Gibco, Ireland) and human recombinant PDGFA (R&D Systems^TM^).

### [^89^Zr] labeling of anti-PD1 antibody

DFO conjugated anti-PD1 antibody was prepared using a reported procedure with modifications (Vosjan et al., 2010). Briefly, 5.0 mg of anti-PD1 antibody was dissolved in 779 µl of PBS, and pH was adjusted to 8.9-9.1 with 80 µl of 0.1 M Na_2_CO_3_. A five-fold molar excess of DFO-Bz-NCS (126 μg in 12.6 μl of DMSO) was added and the resulting solution was incubated for 30 min at 37 °C using a thermomixer at 600 rpm. To remove non-conjugated DFO-Bz-NCS, DFO-PD1 was purified by PD-10 column using PBS. The purified DFO-PD1 conjugate was stored at −80 °C. For ^89^Zr labeling of DFO-anti-PD1 mixture of [^89^Zr]-oxalate (520 µCi in 38 µl of 1 M HEPES, pH 7.4) with 100 µl of PBS and 50 µl (90 µg) of DFO-PD1 in 1.5 ml Eppendorf tube was incubated at 30 °C and 600 rpm for 10 min. The formation of ^89^Zr-DFO-anti-PD1 was monitored by radio-TLC using ITLC-SG strips and 50 mM EDTA (pH 5) as the mobile phase.

### Mice

*CrTac:NCr-Foxn1^nu^* mice (Taconic) referred to in the text as *NCr* were maintained on a normal mouse diet. All animal experiments were conducted according to protocols approved by the Institutional Animal Care and Use Committee of Columbia University Medical Center.

### Intracranial injections

Mouse serum albumin (ALB, MilliporeSigma, MA) or anti-PD-1 antibodies (BE0273, BioCell, NH) were labeled with Cy7 near-infrared fluorescent dye according to manufacturer instructions (Lumiprobe, MD). In some experiments, G48a human glioblastoma cells were labeled with Qtracker 800 according to manufacturer instructions (Molecular Probes, CA). Cy7ALB (5 µl of 2.6 mg/ml in PBS) was injected intracranially 2.5 mm deep into the right cerebral hemisphere 2 mm posterior from bregma and 2 mm to the right of the sagittal suture. One and four days after injection mice were imaged using a 3D optical/CT scanner (MILabs, Netherlands). Parameters were set for Cy7 fluorescence with 20 sec exposure time and excitation at 710 nm and emission at 775 nm. CT was used for morphological references. Images were reconstructed and analyzed using Imalytics ver 2.1 software (MILabs, Netherlands). For orthotopic GBM tumor induction, firefly luciferase-expressing human G48a and mouse GBM cells (1 × 10^5^ cells in 2 µl of DPBS) were intracranially injected 2 mm deep, 2 mm to the right from sagittal suture and 2 mm posterior to the bregma in the right cerebral hemisphere through a scalp incision into 6-weeks old *NCr* mice (Taconic, NY) using a stereotactic instrument (Stoelting, IL) under isoflurane anesthesia (Sattiraju, Xiong, et al., 2017). After implantation, mice were scanned for luciferase activity using a 3D optical/CT scanner (MILabs, Netherlands) for confirmation of tumor growth. Mice with growing human G48a and mouse GBMs were imaged again 3 weeks after implantation; animals with similar luciferase signals were selected and used for BBB integrity studies. Mice with murine GBMs were imaged approximately 4 weeks after tumor cell implantation to confirm tumor growth. Animals with similar tumor signals were selected and randomly divided into two groups. For lipopolysaccharide (LPS) injections 5 µl of 1 mg/ml solution of LPS (MilliporeSigma^TM^, MA) in PBS was injected intracranially 2.5 mm deep into the right cerebral hemisphere 2 mm posterior from bregma and 2 mm to the right from the sagittal suture. Control mice received 5 µl of PBS. The next day LPS-treated and control mice were injected into tail vein with Cy7 labeled albumin (75 μl of 2.6 mg/ml Cy7-albumin per mouse). 3D OI/CT imaging was performed 24 h after Cy7-albumin injection.

### *3D In vivo* optical imaging (OI)/CT

To detect luciferase activity in mice with growing G48a or murine GBMs, animals were injected *ip* with 0.25 ml of 30 mg/ml solution of luciferin (Research Products International Corp, IL) in PBS. The bioluminescent image was acquired under isoflurane anesthesia using MILabs 3D OI/CT imager (MIlabs, Netherlands), as we previously published (Nguyen et al., 2011; Sattiraju, Sai, Xuan, et al., 2017). To detect the intracranial accumulation of Cy7, mice with growing G48a or murine GBMs were injected *iv* with 75 µl of either Cy7 labeled mouse albumin (Cy7ALB) or Cy7 labeled anti PD-1 antibody (Cy7αPD-1). The 3D fluorescent image was acquired under isoflurane anesthesia using MILabs 3D OI/CT scanner 24 h after tracer administrations. Images were reconstructed using MILabs OI-PP ver 1.8 software. In some experiments, 0.3 ml of omnipaque^TM^ 350 (GE Healthcare, IL) was injected intravenously 2–3 min prior to CT acquisition.

### Focused ultrasound with microbubbles

DEFINITY^®^ microbubbles were prepared according to manufacturer instructions (Lantheus Medical Imaging, Inc., Billerica, MA). Focused ultrasound was applied through an intact skull to the right cerebral hemisphere targeting the site of the previously injected GBM cells. Localization of the ultrasound beam with the brain was achieved using stereotactic localization of skull landmarks with a brain atlas-guided focused ultrasound system (RK50, FUS Instruments, Toronto, Canada). For control untreated mice, HIFU was applied to the same spot GBM cells were routinely injected in the right hemisphere (2 mm posterior and 2 mm lateral from the bregma) (Sattiraju, Xiong, et al., 2017). Ultrasound was delivered as a series of burst exposures (10 ms duration, 1 s repetition frequency, ultrasound frequency of 1.5 MHz, 0.5-0.7 MPa peak negative focal pressure). Simultaneous with the start of the HIFU application, DEFINITY^®^ microbubbles (4.5 × 10^7^ microbubbles in 0.5 ml of 0.9% NaCl sterile solution) was infused through the tail vein catheter at a flow rate of 50 µl/min into a mouse tail vein (Szablowski et al., 2018). Immediately after HIFU treatment, 75 µl of Cy7ALB (2.6 mg/ml) or 75 µl of Cy7αPD-1 antibody (2.2 mg/ml) were injected through the same tail vein catheter. In some mice, 100 µCi of ^89^Zr labeled anti-PD-1 antibody ([^89^Zr]- αPD-1) was injected together with Cy7-αPD-1 antibody.

### PET experiments

Luciferase-expressing orthotopic tumors were imaged after *ip* injection of luciferin (30 mg/ml, 0.25 ml/mouse) as described in 3D optical session and screened for luciferase activity. Mice harboring G48a GBMs of a similar size were screened using micro PET with [^18^F]-fluciclovine tracer by injecting *iv* 150 µCi of [^18^F]-fluciclovine and acquiring PET images 1 h after injection. PET images were reconstructed using 3D-OSEM algorithm with 3-iterations in 256 × 256 matrix (Inveon, Siemens, Germany) and analyzed using VivoQuant ver 4 (Invicro, Boston, MA).

### Quantification and statistical analysis

Statistical analysis was performed using Prism 8.0 and SAS 9.4 (SAS Inc., Cary, NC). All data are represented as mean ± standard error. Statistical *p*-values were calculated using two-tailed Student’s *t*-tests for unpaired samples known to be normally distributed and Wilcoxon rank-sum tests for unpaired samples not normally distributed. For paired samples, the changes between two sides (e.g. injected side vs. non-injected side) or the pre- and post-test (e.g. day 1 vs. day 8) are usually normally distributed, so paired *t*-tests were used to calculate the *p*-values. *p*-Values less than .05 were considered as statistical significance.

## Results

### 3D OI/CT localizes GBM cell placement in mice brains

**Figure 2.**
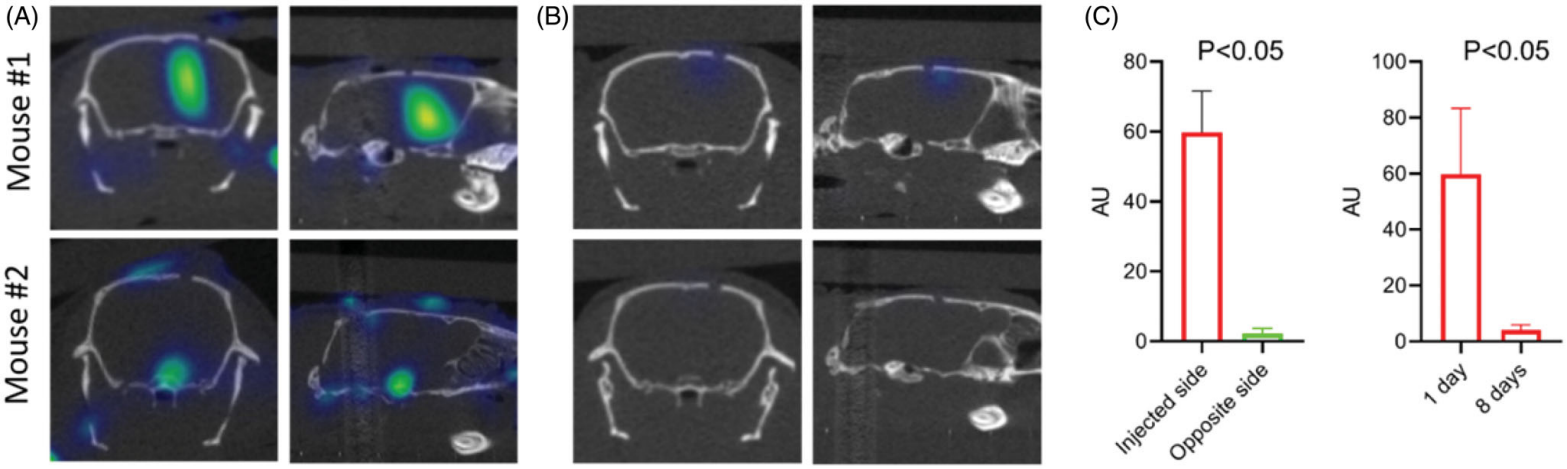
3D optical/CT localization of GBM cells labeled with Cy7. GBM cells were stereotactically implanted intracranially after being labeled with Cy7 and imaged with 3D OI/CT. (A) Coronal and sagittal projections of 3D optical/CT scans of the mouse brains demonstrating successful placement of labeled GBM cells but at different depths, with mouse 2 demonstrating a more inferior placement compared to mouse 1. (B) Same mice as (A) imaged 8 days post cell implantation, demonstrating near complete resolution of signal. (C, left panel) Cy7-fluorescence intensity in injected side versus un-injected side. (C, right panel) Cy7-fluorescence signal disappeared by day 8 after injection.

To determine if 3D OI/CT can be used to track intracranial injections of human GBM cells, we labeled G48a cells with Cy7 and injected them into the right hemisphere under stereotactic guidance. We imaged fluorescent signal 24 h (Figure 2(A)) and 8 days (Figure 2(B)) after cell implantation using 3D OI/CT imaging and compared it to the background fluorescence in the contralateral hemisphere (Figure 2(C), right graph). 24 h after GBM cell implantation, a robust Cy7 signal was observed in the injection area of all treated mice (Figure 2(A)). This Cy7 signal was significantly higher than the background fluorescence in the untreated left hemisphere (Figure 2(C)). Eight days after GBM cell implantation the Cy7 signal diminished to background level (Figure 2(C), right graph).

### 3D OI/CT can detect intracranially injected Cy7 labeled albumin to ensure intracranial delivery and localization after CED

**Figure 3.**
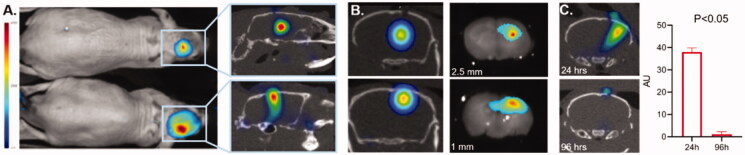
Validation of 3D optical/CT localization of Cy7 labeled mouse albumin (Cy7ALB) directly injected into the brain of a normal mouse. 5 µl of Cy7ALB was injected into right hemisphere. (**A**) Cy7 epi fluorescent images of 2 different mice 24 h after intracranial injection of Cy7ALB showing accumulation on 2D (left) and 3D OI/CT (right), confirming intracranial placement of infused Cy7ALB in the top mouse but superficial localization in the bottom mouse. Note that the intensity on the epi fluorescent images is heavily influenced by how close to the surface the dye is located rather than amount of Cy7ALB present, due to tissue attenuation of the Cy7 signal. (B) Confirmation of localization of Cy7ALB 3D OI/CT on sections of mouse brain imaged post necropsy. Mice were implanted with Cy7ALB at 2.5 mm depth (top, *n* = 3) and 1 mm depth (bottom, *n* = 3) and imaged with 3D OI/CT 24 h after injection (left panels). Immediately after imaging, mice were euthanized and their brains were sectioned and imaged (right panels) to confirm localization of 3D OI/CT images. (C) Coronal 3D OI/CT images of a representative mouse 24 h (top) and 96 h (bottom) after injection of Cy7ALB signal, demonstrating intracranial signal 24 h after injection but not at 96 h, indicating the potential to reimage subsequent infusions. Quantification of the results is shown on the right (*n* = 4).

To demonstrate that 3D OI/CT can be used to validate loco-regional infusions, we labeled mouse albumin with Cy7 dye and administered it intracranially using locoregional delivery. Albumin was selected because we confirmed that it does not cross the BBB (Di Pardo et al., 2017) and is the approximate size of macromolecular therapeutics such as antibodies. We infused mice with Cy7ALB intracranially into the right hemisphere under stereotactic guidance. Mice were infused with Cy7ALB at 2.5 mm depth (*n* = 3) and 1 mm depth (*n* = 3) and imaged with 3D OI/CT. Using 3D OI/CT, we accurately detected and localized infused Cy7 fluorescence inside the brain at the injection site (Figure 3(A)), which we confirmed on post-necropsy brain sections (Figure 3(B) right panels). This contrasts with standard 2D epi-fluorescent imaging with the same scanner that demonstrated a deceivingly higher signal to the more superficially infused Cy7ALB. Importantly, the signal disappeared from the intracranial component 4 days after injection (Figure 3(C)), allowing this technique to be used for repeat injections if needed (*n* = 4). Thus, 3D OI/CT can play a decisive role in confirming proper tumor perfusion of experimental therapeutics after locoregional infusion.

### 3D OI/CT visualizes BBB disruption following systemically administered NIR contrast

**Figure 4.**
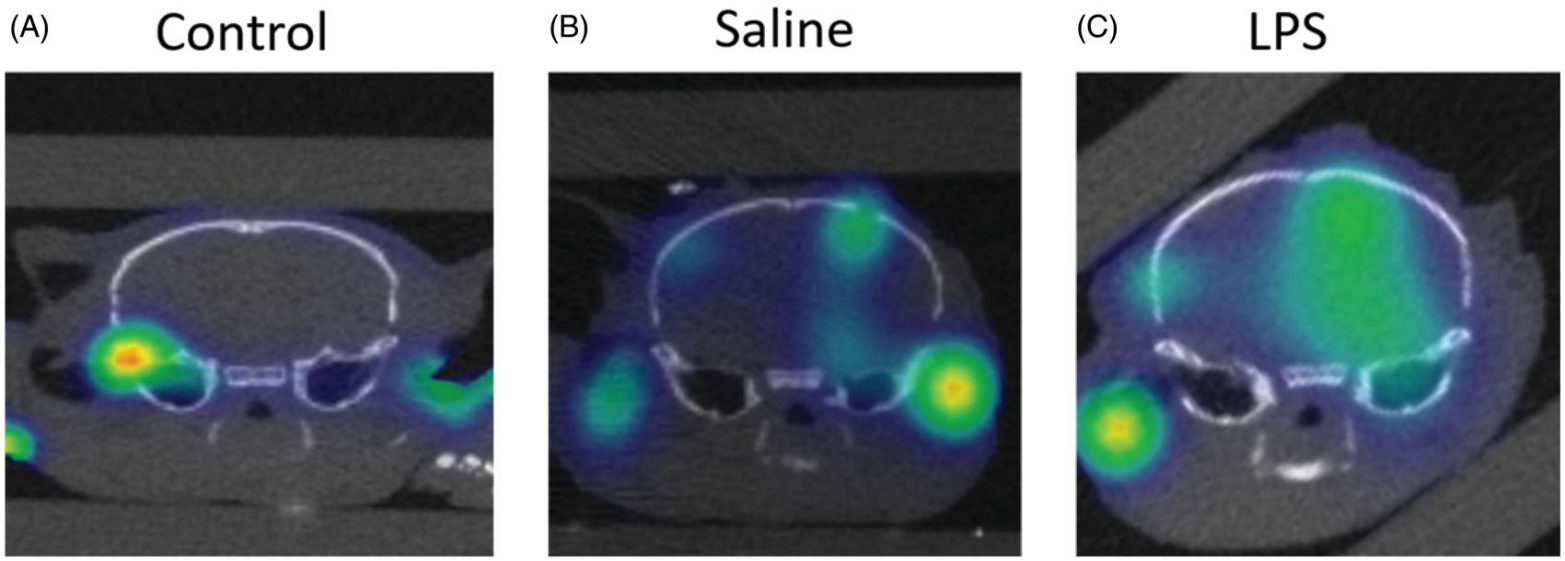
Detection of BBB permeability with systemically injected Cy7ALB. BBB permeability was accomplished using the previously reported intracranial LPS model to induce inflammatory changes. 24 h after stereotactic LPS injection, Cy7ALB was injected IV and imaged 24 h post injection with 3D OI/CT. Coronal images are shown of (A) control with no intracranial injection, (B) control with intracranial injection of saline, and (C) mouse injected intracranially with LPS. Images demonstrate a lack of Cy7ALB in the control brain with an intact BBB, minimal BBB permeability in the saline infused brain and substantial permeability in LPS-infused mouse brain, indicating potential of OI/CT to detect intravenously injected Cy7ALB when the BBB is compromised.

Given the spatial localization capabilities of 3D OI/CT, we tested its ability to detect contrast extravasation following BBB disruption typically seen in GBM. We again used Cy7ALB as optical contrast, as it does not naturally cross the BBB (Figure 4(A)). We initially used an LPS BBB permeability model (Espinosa-Oliva et al., 2013) for proof-of-concept experiments. LPS induces significant inflammation and has been shown to permeabilize the BBB after intracranial administration. We injected LPS intracranially and compared 3D OI/CT images of LPS-infused mice (*n* = 4) against normal controls (*n* = 4) or mice injected with saline alone (*n* = 4), which we and others have shown minimally permeabilizes the BBB in the first few days after needle truama of an injection (Sattiraju, Sai, & Mintz, 2017). 24 h post LPS injection, we systemically injected Cy7ALB via the tail vein and performed 3D OI/CT. Images demonstrated the most significant increase of Cy7 signal along the injection track in the LPS mice whereas the saline-injected mice demonstrated minimal signal due to mechanical damage from the needle (Figure 4). In contrast, control mice without any injection demonstrated no intracranial optical signal, validating our method to detect and localize BBB permeability when the BBB is compromised (Figure 4).

### 3D OI/CT with Cy7ALB contrast can be used to detect GBMs in rodent models

**Figure 5.**
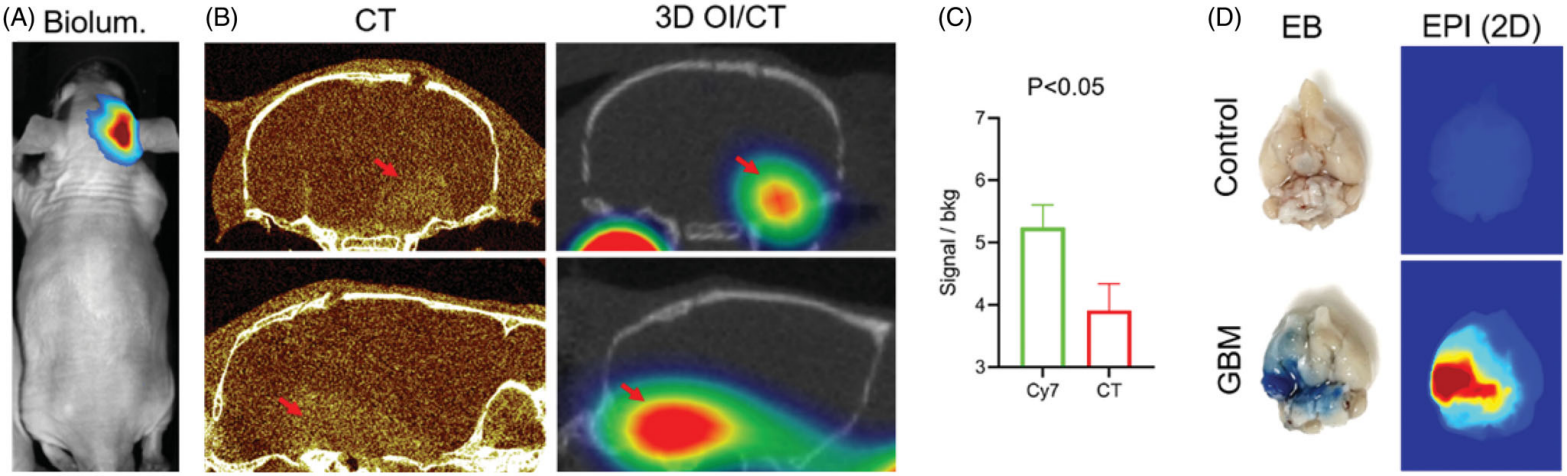
3D optical/CT visualization of glioblastoma in a mouse brain using systemically injected Cy7ALB. (A) Bioluminescence (biolum) image of mouse with implanted G48a-FLuc cells. (B) Coronal (top) and sagittal (bottom) views of microCT with Omnipaque^TM^ contrast (left panels) and 3D optical/CT (right panels) 24 h post systemic injection of Cy7ALB. Images demonstrate intracranial Cy7ALB accumulation in the mouse with growing G48a GBM caused by BBB permeability in the region of the tumor, corresponding to the CT contrast accumulation. (C) Tumor to background ratio obtained using Cy7-albumin 3D optical detection (Cy7ALB) and CT with contrast (CT). (D) After imaging, mice were injected with Evan’s blue (EB) dye and euthanized 4 h later to confirm BBB penetration. Photograph of EB and epi fluorescent images of the post mortem brain of a control non tumor bearing mouse (top) along with brain of the GBM-bearing mouse from images A and B (bottom), confirming BBB permeability and Cy7ALB accumulation, respectively.

MRI with gadolinium contrast is commonly used to detect GBMs based on their central BBB permeability. In order to test if Cy7ALB 3D OI/CT can be used as an alternative to detect contrast enhancement of intracranial GBMs, we grew orthotopic GBMs by implanting human G48a GBM cells (Figure 5) or murine GBM cells (Figure S1). Following tumor growth using bioluminescence imaging (Figure 5(A)), mice were further imaged to localize their intracranial GBMs using contrast-enhanced microCT (Figure 5(B)) (*n* = 4) or PET/CT (Figure S1). We simultaneously injected mice systemically via the tail vein with Cy7ALB and imaged with 3D OI/CT (*n* = 5). Similar to the contrast enhancement of the GBM on microCT, we detected the accumulation of Cy7ALB in the brain following iv injection (compare left and right panels in Figure 5(B)). Intracranial Cy7ALB fluorescent signal was localized in the same area enhanced by the contrast on microCT, but with a significantly higher tumor-to-background ratio (Figure 5(B,C)). In order to verify intracranial Cy7ALB localization through disrupted BBB, we injected mice with Evan’s blue (EB) dye (Bullard & Bigner, 1984; Goldim et al., 2019) and subsequently euthanized and imaged their dissected brains to visualize the EB and Cy7ALB distribution using light microscopy and epifluorescence, respectively (Figure 5(D)). We found that only mice inoculated with GBM cells that showed luciferase signal *in vivo* and EB signal *in vitro*, demonstrated Cy7ALB signal in brain tissue. We found a similar ability to use 3D OI/CT to detect GBM in an independent model of murine GBM (Figure S1). These data validate that 3D OI/CT can be used to image GBMs that exhibit contrast extravasation.

### 3D OI/CT with Cy7ALB contrast confirms BBB opening by HIFU

**Figure 6.**
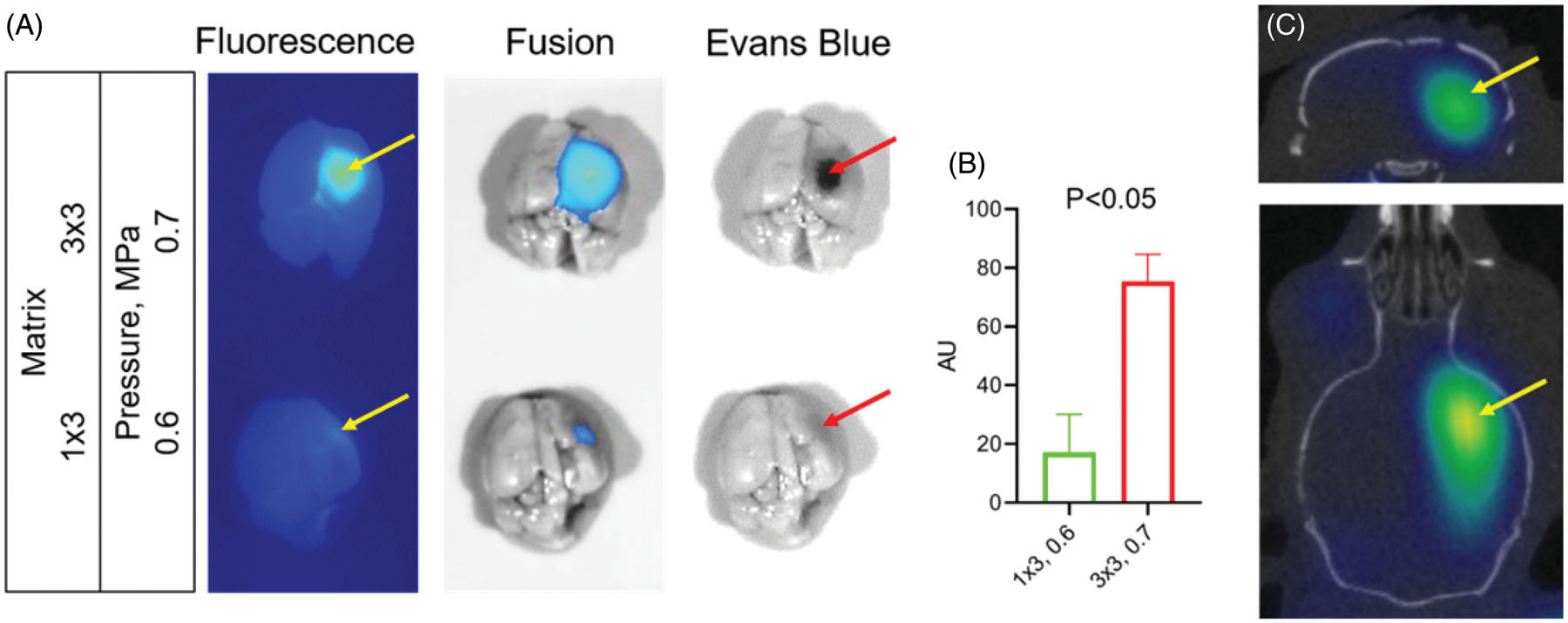
Detection of blood–brain barrier (BBB) permeability after high intensity focused ultrasound (HIFU). (A) Images of dissected brains from mice treated with HIFU pressure at 0.7 (top) and 0.5 (bottom) MPa. Following HIFU, animals were immediately injected IV with a mixture of Cy7ALB (yellow arrows) and Evan’s blue (red arrows). Brains were dissected 24 h after treatment. Note the considerably stronger Cy7 fluorescence and Evan’s blue staining in the brains of mice subjected to HIFU at 0.7 power compared to 0.6. (B) Quantification of Cy7ALB in brains of HIFU treated mice. (C) 3D OI/CT image of Cy7ALB accumulation (yellow arrow) in the brain of mouse 24 h after HIFU treatment shown in coronal (top) and axial (bottom) projections.

HIFU with microbubbles has shown the potential to disrupt the BBB for systemic therapeutic delivery. To validate the relationship between iv injected Cy7ALB accumulation in the brain and HIFU treatment, we treated mice (*n* = 3 for each condition) with varying matrix sizes and pressures in combination with microbubbles. We subsequently injected mice with a mixture of EB and Cy7ALB via the tail vein, euthanized the animals, dissected brains, and imaged with epifluorescence imaging. We found that HIFU induced Cy7ALB accumulation correlated with the HIFU treatment regiments (pressure level and matrix size) and matched the distribution of EB dye (Figure 6(A,B)). HIFU at a higher power level (0.7 vs 0.6 MPa) and at larger affected area (3 × 3 vs 1 × 3 points matrix) regiment induced considerably greater Cy7 accumulation in the brain compared to the lower power/smaller matrix regiment (compare top and bottom panels on Figure 6(A)). As expected, no Cy7 fluorescence was observed in the brains of untreated mice. Thus, systemically administered Cy7ALB correlated with the amount of HIFU-induced BBB permeability and could be used as a contrast for our subsequent HIFU 3D OI/CT experiments. We injected mice iv with Cy7ALB and imaged using 3D OI/CT 24 h after HIFU treatment (Figure 6(C)). We found that 3D OI/CT images confirmed precise localization of Cy7 fluorescence in the right hemisphere in the area targeted by the HIFU application (Figure 6(C)). This demonstrates that 3D OI/CT with Cy7ALB contrast has the potential as an optical probe to verify HIFU-induced opening of the BBB prior to systemic therapeutic injections.

### 3D OI/CT confirms GBM targeting of systemically administered antibody treatment following HIFU-mediated BBB disruption

**Figure 7.**
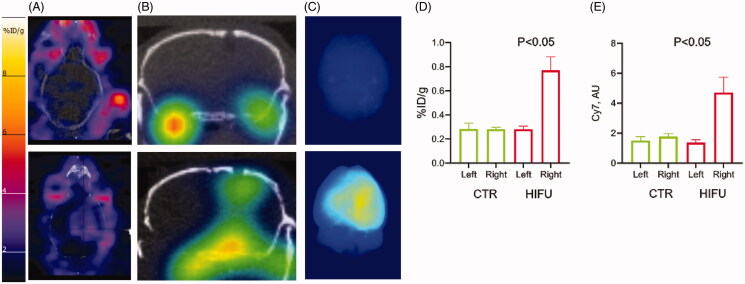
Systemic therapeutic delivery through HIFU induced BBB permeability using PET/CT and optical/CT. Mice were treated with HIFU with microbubble infusion and immediately injected IV with Cy7-anti-PD1 and [^89^Zr]-anti-PD1 antibodies (see methods for details, bottom panels). Control mice were not exposed to HIFU (top panels). (A) 30 min static PET/CT images were obtained 24 h after injection and shown in axial projection. (B) After PET/CT images were aquired, mice were scanned with 3D optical/CT imaging to detect intracranial Cy7-anti-PD1 antibody accumulation, shown in coronal projection. (C) Immediately after 3D optical/CT scaning brains were dissected and both PET and epifluorescent optical images were taken. PET (D) and optical (E) uptake were quantitated and analyzed for statistical significance.

While high molecular weight targeted antibodies and proteins are showing promise against many systemic malignancies, they are limited in the fight against GBM due to their exclusion by the BBB. In GBM, it is hoped that increasing the concentration of therapeutic antibodies using HIFU to permeabilize the BBB can successfully target GBM biomarkers or activate the endogenous immune system. In order to test the potential of using 3D OI/CT for confirmation of brain tumor perfusion with systemically administered antibodies, we labeled anti-PD-1 (αPD-1) antibodies with Cy7 and ^89^Zr. Immediately after HIFU treatment, animals were injected with a mixture of Cy7-αPD-1 and [^89^Zr]αPD-1 labeled antibodies. ^89^Zr is a positron-emitting isotope that can be detected using PET/CT imaging. In the control animals not treated with HIFU (*n* = 4), neither 3D OI/CT nor PET/CT detected significant Cy7 or ^89^Zr labeled αPD-1 antibody (upper panels in Figure 7(A,B)). In contrast, HIFU treatment (*n* = 4) produced a focal opening of the BBB allowing for αPD-1 antibody entry and accumulation in the brain, which was detected via 3D OI/CT and microPET imaging (lower panels on Figure 7(A,B)). Both PET and 3D OI/CT demonstrated significantly (*p* < 0.05) higher accumulation of [^89^Zr]αPD-1 (Figure 7(D)) and Cy7-αPD1 (Figure 7(E)) in the brains after HIFU compared to controls. To confirm our *in vivo* findings, we dissected the brains of mice and imaged them *ex vivo* (Figure 7(C)). We found that the region of accumulation of Cy7-αPD-1 corresponded to the area of HIFU application (lower panel in Figure 7(C)). As expected, Cy7-αPD-1 was not detected in the brains of mice not treated with HIFU (upper panel in Figure 7(C)). Importantly, we found both *in vivo* and *in vitro*, that there are variances in the effectiveness of HIFU between animals, which can be quantified using 3D OI/CT and PET. This can prove critical when assessing efficacies of treatments after using HIFU to permeabilize the BBB. Both PET and 3D OI/CT techniques showed similar results of the αPD-1 antibody accumulation, which indicates that 3D OI/CT can serve as a preclinical surrogate for PET imaging of drug distribution.

## Discussion

The BBB serves as a transport barrier and is crucial for the brain homeostasis and preventing toxins from entering a central nervous system (Daneman & Prat, 2015). GBMs typically disrupts the BBB in a number of ways, such as mechanical breakdown, downregulation of tight junction proteins, and redistribution of astrocyte potassium channels (Zhang et al., 1992; Liebner et al., 2000; Wolburg et al., 2003; Papadopoulos et al., 2004; Warth et al., 2004; Watkins et al., 2014). *In vivo* imaging of GBM using contrast-enhnced MRI relies on this BBB permeability that is induced naturally by the tumor (Michiwaki et al., 2019). In this work, we validated 3D OI/CT as a means of imaging the BBB permeability of GBMs using Cy7ALB contrast enhancement to localize GBMs (Figure 5).

Infiltrating cells are found several centimeters outside enhancing tumor margin and away from the tumor-induced BBB breakage (Spiteri et al., 2019). Thus, the intact BBB around infiltrating tumor cells remains an obstacle that blocks or limits intracranial delivery of the therapeutics to the brain, especially for large macromolecules such as IgG (Zlokovic et al., 1990; Wevers et al., 2018). HIFU in combination with systemically injected microbubbles applied to regions of the tumor that do not demonstrate contrast enhancement is a promising technique for noninvasively inducing transient BBB opening for intracranial drug delivery (Park et al., 2012; Yang et al., 2012; Carpentier et al., 2016; Kobus et al., 2016; Sattiraju, Sun, et al., 2017; Arvanitis et al., 2018). HIFU application for the facilitated delivery of therapeutic macromolecules across the BBB has shown promise in early-stage clinical trials (Mazerolle et al., 2019). This work establishes 3D OI/CT in conjunction with Cy7-labeled contrast as a methodology of localizing BBB permeability. For HIFU applications aiming to deliver protein-based therapeutics, the advantage of albumin contrast agents over the traditional low molecular weight radiological contrast agents is that it may more accurately represent the distribution of protein macromolecules relevant to possible immunoglobulin-based treatments, such as immunotherapies (Wan et al., 2019). In our work, we demonstrate the potential use of 3D OI/CT in 3 different preclinical models of BBB permeability (LPS, GBM, and HIFU + microbubbles) (Figures 4–7). We also showed the longevity (over 24 h) of Cy7ALB in brain tissue following initial intravenous administration, which is more relevant for the evaluation of immunoglobulin therapeutic applications compared to small molecules that are typically cleared in a few hours.

We demonstrated that we can use 3D OI/CT to directly label a prototype antibody therapy with Cy7 and image its distribution after HIFU-mediated BBB disruption (Figure 7) as a monitoring tool to measure the delivery of therapeutic proteins to the tumors through the permeabilized BBB. This is highly clinically relevant when testing the preclinical efficacy of potential macromolecular therapeutic strategies such as immunotherapy. Importantly, 3D OI/CT can act as an accessible preclinical surrogate for using radiolabeled entities in future clinical testing.

CED delivers therapeutics directly to the brain tumor and surrounding brain under constant pressure. A critical lesson learned from the initial clinical trials using CED to deliver novel cytotoxins to GBM is that it is essential to confirm that the drug-infused via CED successfully covers the tumor and surrounding area (Sampson et al., 2010). We therefore, validated 3D OI/CT as a scalable preclinical methodology to confirm infusion location and coverage. This becomes a foundation of drug development because the efficacy signal will not be indicative of the drug’s potency unless proper delivery can be confirmed. We demonstrated that we can localize injections in the brain to determine if the infused drug properly covers the tumor (Figure 3). Furthermore, our ability to label therapeutics directly with Cy7, as we demonstrated with antibodies (Figure 7), gives a very precise picture of the actual drug distribution, which is more direct than a contrast surrogate such as gadolinium (for MRI).

In conclusion, the use of 3D OI/CT for spatial reference of locoregionally delivered drugs, cells, and HIFU-mediated BBB permeability creates the possibility for its use as a preclinical tool for drug development. Future experiments will involve using 3D OI/CT as a tool for the inclusion of tumor-bearing mice in larger therapeutic studies and correlation of 3D OI/CT signal after CED or HIFU with survival after therapeutic intervention.
